# Supplementary material for: Baseline Characterization of the Gut Microbiota of Field and Colony Populations of Phlebotomus tobbi and Preliminary Assessment of the Anti-Leishmanial Activity of Cultivable Bacteria
Source: Pathogens. 2026 Jun 23;15(7):658. doi: 10.3390/pathogens15070658 (PMC13416292; doi:10.3390/pathogens15070658)
Supplement: Supplementary file 1 [file pathogens-15-00658-s001.zip › pathogens-4357168-File S1/Taxa2ASV_Decomposer/Family/f__Staphylococcaceae/abun.plot.top12.pdf]

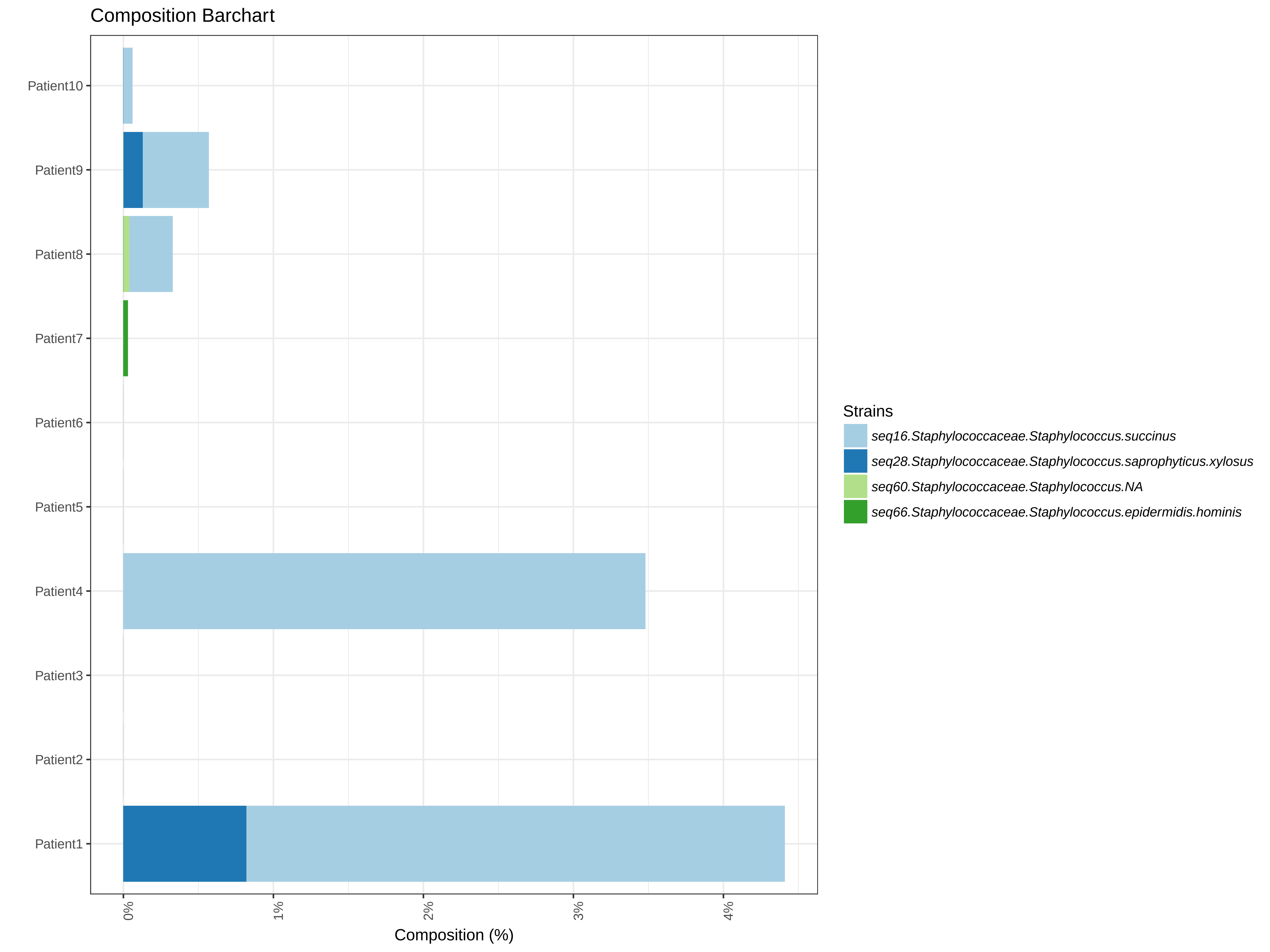

Composition Barchart
Patient10
Patient9
Patient8
Patient7
Patient6
Patient5
Patient4
Patient3
Patient2
Patient1
0%
1%
2%
3%
4%
Composition (%)
Strains
seq16.Staphylococcaceae.Staphylococcus.succinus
seq28.Staphylococcaceae.Staphylococcus.saprophyticus.xylosus
seq60.Staphylococcaceae.Staphylococcus.NA
seq66.Staphylococcaceae.Staphylococcus.epidermidis.hominis
